# Incidence of Neuralgic Amyotrophy (Parsonage Turner Syndrome) in a Primary Care Setting - A Prospective Cohort Study

**DOI:** 10.1371/journal.pone.0128361

**Published:** 2015-05-27

**Authors:** Nens van Alfen, Jeroen J. J. van Eijk, Tessa Ennik, Sean O. Flynn, Inge E. G. Nobacht, Jan T. Groothuis, Sigrid Pillen, Floris A. van de Laar

**Affiliations:** 1 Department of Neurology, Donders Centre for Neuroscience, Radboud university medical centre, Nijmegen, The Netherlands; 2 Department of Neurology, Jeroen Bosch Hospital, Den Bosch, The Netherlands; 3 Department of Neurology, Erasmus Medical Center, Rotterdam, The Netherlands; 4 Royal College of Surgeons in Ireland, Dublin, Ireland; 5 Primary Health Centre Oosterhout, Nijmegen, The Netherlands; 6 Department of Rehabilitation, Donders Centre for Neuroscience, Radboud university medical centre, Nijmegen, The Netherlands; 7 Department of Neurology, Canisius Wilhelmina Hospital, Nijmegen, The Netherlands; 8 Department of Primary and Community Care, Radboud Institute for Health Sciences, Radboud university medical centre, The Netherlands & Academic Health Centre Thermion, Lent, The Netherlands; University of Würzburg, GERMANY

## Abstract

**Objective:**

Neuralgic amyotrophy is considered a rare peripheral nervous system disorder but in practice seems grossly under recognized, which negatively affects care for these patients. In this study we prospectively counted the one-year incidence rate of classic neuralgic amyotrophy in a primary care setting.

**Methods:**

In a prospective cohort study during the year 2012 we registered all new cases of neck, shoulder or arm complaints from two large primary care centers serving a population of 14,118. Prior to study, general practitioners received a short training on how to diagnose classic neuralgic amyotrophy. Neuralgic amyotrophy was defined according to published criteria irrespective of family history. Only patients with a classic phenotype were counted as definite cases. After inclusion, patients with suspected neuralgic amyotrophy who had not yet seen a neurologist were offered neurologic evaluation for diagnostic confirmation.

**Results:**

Of the 492 patients identified with new onset neck, shoulder or arm complaints, 34 were suspected of having neuralgic amyotrophy. After neurologic evaluation the diagnosis was confirmed in 14 patients. This amounts to a one-year incidence rate for classic neuralgic amyotrophy of 1 per 1000.

**Conclusions:**

Our findings suggest that neuralgic amyotrophy is 30-50 times more common than previously thought. Unawareness of the disorder and its clinical presentation seems the most likely explanation for this difference. An incidence rate of 1 per 1000 and the long-term sequelae many patients suffer warrant more vigilance in diagnosing the disorder, to pave the way for timely treatment and prevent complications.

## Introduction

Neuralgic amyotrophy (also called Parsonage Turner syndrome or brachial plexus neuritis) is a distinct peripheral nervous system disorder, characterized by one or more episodes of acute, severe pain in the upper extremity, quickly followed by a multifocal paresis [1]. Recovery is usually slow over the course of one to two years, with a large subset of patients at risk for developing long term complaints [2–4]. Recognition of the disorder as a distinct clinical entity has proved challenging for primary care physicians and neurologists, especially with the various clinical phenotypes within the spectrum of neuralgic amyotrophy, such as painless episodes and episodes that involve the lower brachial plexus or other peripheral nerves [1]. However, in about 70% of the patients a classic, easily recognizable, uniform phenotype is found with acute pain in the shoulder region followed by scapular winging, weakness of shoulder abduction and exorotation, diminished pinch grip and forearm pronation weakness.

As it is likely that neuralgic amyotrophy attacks are ultimately induced by an auto-immune response to the peripheral nervous system [1–5], efforts have been undertaken to attempt treatment in the acute phase with immunomodulants such as corticosteroids or iv gammaglobuline [6–11]. Unfortunately, a randomized controlled trial of corticosteroids failed because it proved impossible to recruit the needed number of participants within the three-year trial period. The main cause was the delay in diagnosis after symptom onset [NvA, unpublished observation], consistent with a previous cohort study with a median time to diagnose neuralgic amyotrophy of 11 weeks [2].

The estimated incidence of neuralgic amyotrophy as found in a primary care region and hospital setting was 1–3 per 100.000 per year [12,13]. The Radboud university medical center has hosted a national expert center for neuralgic amyotrophy in the Netherlands for the past 15 years. However, from our clinical experience and from the number of new cases we see in our hospital personnel [2] we suspect the true incidence rate to be more likely in the order of 1 per 1000 per year. Prompt recognition of the disorder would enable research of the appropriate acute treatment and likely prevent some of the common long-term sequelae [3,4].

To study the incidence rate of the classic neuralgic amyotrophy phenotype in the general population, a collaboration was initiated with two large primary care practices allied with our university medical center. A second objective was to study the ratio and characteristics of neuralgic amyotrophy to that of other disorders of the neck, shoulder or arm in a primary care setting.

## Materials and Methods

The two participating primary care centers in this prospective cohort study were located in the suburban Nijmegen area. Both centers have been digitally registering diagnostic codes for decades [14]. Patient visits cannot be completed without entering such a code, minimizing the chance of incomplete sampling. For the current study all patients who presented to one of the participating general practitioners between January 1st and December 31st of 2012 with a new episode of complaints of the neck, shoulder(-s) or arm(-s) were entered in the study. To this end, every patient with a specific diagnostic International Classification of Primary Care code (ICPC; Dutch version [15]) indicating neck or upper extremity complaints was extracted from the electronic patient records in five incremental data samples during the study year 2012. The 12 specific ICPC codes used for the searches are shown in Table 1. Only patients whose visit was for a new episode with complaints within the study period were entered in the study sample. As some codes could also indicate complaints in other body regions, only patients with these codes who had symptoms of the neck, shoulders and/or arms were included. Patients who presented with carpal tunnel syndrome were excluded. Details of patients were entered into a database (IBM SPSS Statistics v20.0, IBM Corporation New York USA; data are available as S1 Datafile). Double entries were extracted post-hoc.

**Table 1:** ICPC codes used for patient identification in this study.

L01	neck symptom / complaint
L04	chest symptom / complaint
L08	shoulder symptom / complaint
L09	arm symptom / complaint
L18	muscle pain
L83	neck syndrome
L83.1	cervical disk herniation
L92	shoulder syndrome / frozen shoulder
N18	paralysis / weakness
N94	peripheral neuritis / neuropathy
N94.3	thoracic outlet syndrome
N99	neurological disease / other

To improve the diagnostic capability of the general practitioners for clinically diagnosing classic neuralgic amyotrophy, two one-hour instruction sessions were provided by an experienced neurologist (NvA) and a physical therapist. The differentiating signs between neuralgic amyotrophy and other disorders of the neck or upper extremity with similar symptoms were discussed [16]. General practitioners were instructed to examine patients with a bare upper body (a bra could be left on), with inspection of the shoulders for scapular (a-) symmetry and signs of muscle atrophy. Patients were instructed to abduct both arms simultaneously to the maximum height attainable and then anteflect them again in a slow but deliberate motion. When abnormalities such as scapular winging or scapulothoracic dyskinesia were suspected, this movement was filmed on with a smartphone camera for later review. When a periscapular neurologic abnormality was suspected, additional strength testing of at least the serratus anterior muscle, shoulder exorotation and pinch grip was performed. General practitioners were asked to consider the diagnosis of classic neuralgic amyotrophy when a patient presented with symptoms depicted in Table 2.

**Table 2:** Presenting symptoms and exclusion criteria for considering neuralgic amyotrophy as a diagnosis.

General practitioners were asked to consider the diagnosis when patients presented with:
• New onset shoulder pain (uni- or bilateral)
• NRS pain score of ≥7 on a scale of 0–10
• Abnormal shoulder movement (glenohumeral and/or scapulothoracic) during maximum abduction/anteflexion movement
• When first seen ≥ 3 weeks after onset: paresis of long thoracic nerve, suprascapular nerve, anterior interosseus nerve
Optional signs and symptoms:
• Less severe initial pain with otherwise typical clinical multifocal distribution of weakness and monophasic course
• More extensive multifocal paresis of upper extremity (-ies)
• Asymmetric involvement of other upper extremity
• Areas of vital hypesthesia and/or paresthesia in the upper extremity
• Involvement of other peripheral nerves: lumbosacral plexus, phrenic, recurrent laryngeal nerve
Neuralgic amyotrophy was excluded when patients had:
• Progression of pain and/or weakness > 3 months (except for pain associated with abnormal compensatory shoulder movements)
• Only passive range of motion constraints in the glenohumeral joint
• Horner syndrome
• Perfectly symmetric weakness distribution
• Diabetes mellitus

As our study aimed to reflect incidence in a general population with a standard level of care, we chose not to require that every patient was referred for specialist evaluation. It was the general practitioner who decided whether this was clinically necessary. As neuralgic amyotrophy is primarily a clinical diagnosis, additional investigations (such as electromyography or magnetic resonance imaging) were only considered and offered to the patients when their results would have clinical consequences for their management. After the study inclusion period, patients in whom neuralgic amyotrophy was suspected by their general practitioner but who had not been sent for neurological evaluation initially, were offered a consultation with one of the study neurologists (NvA, JvE, SP) to confirm or outrule the diagnosis. Criteria used by the study neurologists for the clinical diagnosis of classic neuralgic amyotrophy are modified from the criteria for hereditary neuralgic amyotrophy [17,18] that led us to the following case definition: 1. (Sub)acute onset, 2. Initial pain with numerical rating scale (NRS) score ≥ 7, 3. Multifocal distribution mainly in the upper brachial plexus with winged scapula 4. Monophasic course, with slow recovery, 5. Preceding trauma, malignancy, diabetes mellitus and radiation excluded. Of note, patients with less severe or no pain at onset but a phenotype and course that fitted neuralgic amyotrophy were clinically treated as neuralgic amyotrophy, but registered as "probable" cases. Also, when the clinical information was insufficient to reach a diagnosis of neuralgic amyotrophy according to the criteria and no additional information could be obtained, we chose not to include the case as "definite" but as "probable".

### Ethics statement

The Radboud university medical center committee on human research judged that our study was exempt from formal approval. All patients referred to a neurologist because of participating in this study were asked and gave their written informed consent, documented in their electronic patient record. If children would have been referred, consent would have been obtained from the parents or legal custodians. The Radboud university medical center committee on human research made no recommendations on how to record the patients' consent. The authors NvA, TE, JvE, IN, SP and FvdL interacted with the patients during the study. Raw patient data was extracted by NvA, TE and SF and anonymized prior to further analysis. The primary data file for this study (S1 Datafile) was anonymized by removing patients' names and birthdates.

## Results

The two primary care practices in the suburban Nijmegen area had adherent populations of 14118 people as of June 2012. During the study year of 2012, 492 patients with new onset neck, shoulder or arm complaints were identified, making for a total incidence of these complaints of 35 per 1000.

During the year 2012, 34 patients (62% male) were suspected of having neuralgic amyotrophy. The initial ICPC codes (Table 1) used by general practitioners to classify these 34 patients were most commonly L08 (“shoulder complaints”; 15 cases), N94 (“other PNS neuropathy”; 9 cases) and L83 (“cervical complaints”; 4 cases); other codes used were L09, L92 and others (6 cases combined). This variation in coding practice was most likely caused by the initial diagnostic uncertainty.

**Fig 1:**
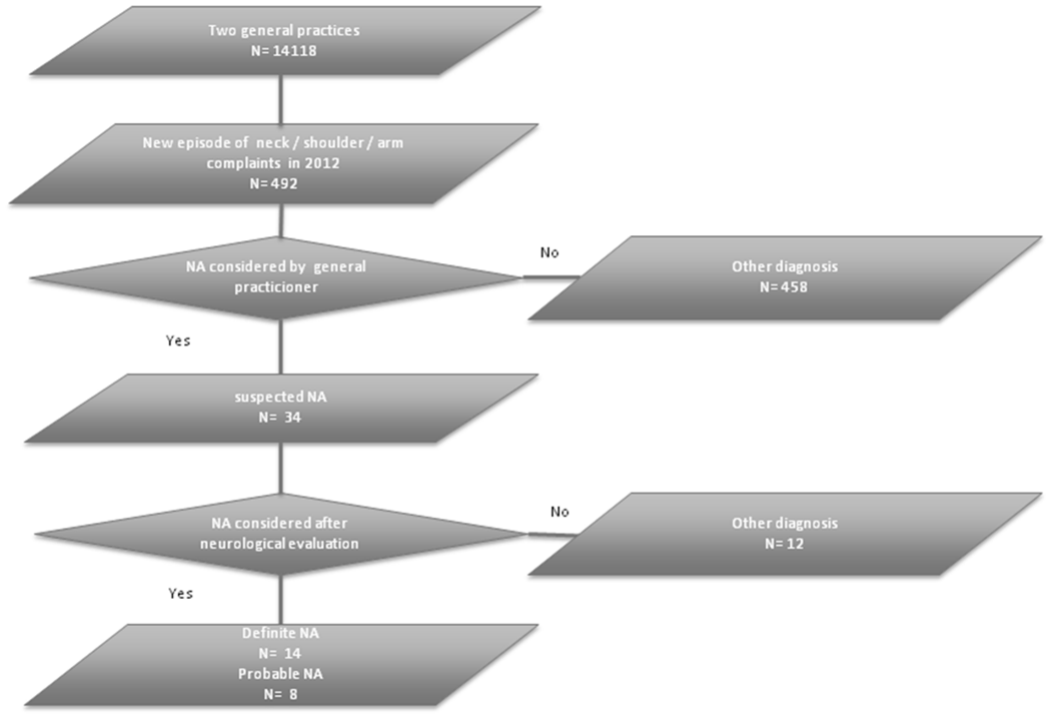
Study flowchart. NA = neuralgic amyotrophy.

After multidisciplinary review of the 34 cases with suspected neuralgic amyotrophy, 14 patients (9 male) were identified with definite neuralgic amyotrophy (Fig 1; for clinical details of these cases see S1 Case Descriptions). Of these 14 patients, 11 were treated with analgesics in acute phase and 8 were treated with high dose oral prednisolone, the latter with good effect in 3, partial relief in 2 and no effect in the remaining 3 patients. Nine patients were started on regular physiotherapy, with good effect in 2, some effect in 1, no effect in 5 and worsening of symptoms in 1. On final follow up, 2/14 patients made a good recovery. We encountered one patient with hereditary neuralgic amyotrophy; a finding compatible with the reported ratio of hereditary versus idiopathic neuralgic amyotrophy of about 1 to 10 [1]. Eight additional patients (six male) were identified who probably had neuralgic amyotrophy but who did not fulfill the pain score criteria at onset (N = 3), in whom the diagnosis could not be further confirmed clinically (N = 2), or who were unavailable for diagnostic confirmation by the neurologist afterwards (N = 3). In the remaining 12 patients neuralgic amyotrophy had been suspected, but the diagnosis was outruled after neurological review. Counting only the 14 definite cases this makes for an incidence rate of 1 per 1000 per year (14 cases in 14,118 people) for classic neuralgic amyotrophy.

For the 470 patients who did not have definite or probable neuralgic amyotrophy the most common diagnoses by ICPC code were L08 (n = 158), L01 (n = 112), L92 (n = 74) and L09 (n = 53). Specific cervical radicular pathology (code L83) was much less often coded (n = 14); we presume that because of diagnostic uncertainty at the first visit these patients were most likely coded as L01 "neck complaints". A residual category was formed by other PNS neuropathies of the upper extremity (n = 7) and the combined other diagnoses (N18, L18 and N99; n = 52). Demographics and symptoms of the three patients groups and their differences can be found in Table 3.

**Table 3:** Demographics and symptoms distribution between patient groups.

Parameter		No NA (n = 470)	Probable NA (n = 8)	Definite NA (n = 14)	Chi^2^ p-value
		N (percentage)	N (percentage)	N (percentage)	
Sex	male	202 (57%)	6 (75%)	9 (64%)	0.06
	female	268 (43%)	2 (25%)	5 (36%)	
Onset age (years)	median	44	46	41	0.37
	range	0–101	31–59	30–69	
Affected side	left	139 (33%)	3 (38%)	4 (28%)	0.84
	right	161 (38%)	4 (50%)	5 (36%)	
	bilateral	119 (29%)	1 (12%)	5 (36%)	
Pain at onset	yes	437 (97%)	7 (88%)	14 (100%)	0.23
	no	13 (3%)	1 (12%)	0 (0%)	
Pain distribution		94 (45%) neck	2 (40%) trapezius	6 (43%) lateral arm	**0.00**
		32 (15%) glenohumeral joint	1 (10%) glenohumeral joint	5 (36%) trapezius	
		19 (9%) lateral arm	1 (10%) scapula	1 (7%) scapula	
		15 (7%) trapezius	1 (10%) lateral arm	1 (7%) glenohumeral joint	
		49 (23%) other	3 (30%) missing	1 (7%) whole arm	
Weakness on physical exam	yes	8 (2%)	5 (63%)	14 (100%)	**0.00**
	no	462 (98%)	3 (27%)	0 (0%)	
Limited glenohumeral range of motion	yes	111 (24%)	1 (13%)	6 (43%)	0.19
	no	359 (76%)	7 (87%)	8 (57%)	
Sensory symptoms	yes	63 (13%)	6 (50%)	8 (57%)	**0.00**
	no	407 (87%)	6 (50%)	6 (43%)	
Preceding event	yes	165 (80%)	6 (75%)	6 (43%)	0.11
	no	42 (20%)	2 (25%)	8 (57%)	
Type of preceding event		80 (49%) strain	2 (25%) strain	2 (33%) illness	**0.00**
		59 (36%) trauma	1 (13%) surgery	2 (33%) strain	
		16 (10%) stress	3 (62%) missing	1 (17%) childbirth	
		10 (5%) other		1 (17%) surgery	

## Discussion

This study of a prospective one-year case count in two large primary care practices demonstrated an incidence rate of classic neuralgic amyotrophy of 1 per 1,000 per year. That is about two orders of magnitude higher than the incidences of 2–3 per 100,000 per year previously reported [12,13]. Given the unfamiliarity of many physicians with the diagnosis, the most likely explanation for this incidence “increase” seems to be under recognition of the disorder and hence the frequent lack of its incorporation in the differential diagnosis of new onset upper extremity complaints. It could also be that the current perception of neuralgic amyotrophy as a rare disorder contributes to self-propagation of this under recognition (e.g. “as neuralgic amyotrophy is so rare, this patient probably does not have it”) [16].

Other factors might also explain the difference, such as a population or environmental variance. There are only two previous studies that looked at the incidence of neuralgic amyotrophy. The first [12] reports a retrospective hospital cohort analysis of 579 cases with possible brachial plexopathy, in which the authors could only confirm neuralgic amyotrophy in 11 cases. This led to a calculated incidence of 1·64 per 100,000 per year. However, the authors also describe a group of 61 cases who had the typical pain but in whom no weakness was found on chart review. As a detailed exam of all upper extremity muscles is clinically challenging and periscapular instability is often overlooked [19], it is possible that a significant number of these patients might have in fact suffered from neuralgic amyotrophy. As a study reporting from a hospital setting, it might also have missed those patients never referred for secondary care. The second study prospectively counted all neurological disorders from 13 primary care practices serving a population of 100,230 in the urban London area [13] and found an incidence rate of 3 per 100,000. As these results also come from a primary care setting in an urban northern European area, the population appears quite similar to that of our current study. This makes it unlikely that there is a population variance that is able to explain a 30-50-fold incidence difference.

The ratio of the incidence of classic neuralgic amyotrophy to that of other disorders in this study was 3%. This means that roughly one in every 33 patients with new onset neck, shoulder or arm complaints in a primary care setting will have neuralgic amyotrophy. In comparison, 47% or one in two patients were diagnosed with musculoskeletal shoulder pathology (codes N08 or L92) in this study, making this 15 times as common a cause as neuralgic amyotrophy.

An incidence of 1 per 1,000 per year means that it is questionable whether neuralgic amyotrophy can still be classified as a rare disease, which is defined as affecting 1 in 2,000 people (i.e. prevalence) or less [20]. In the Netherlands, an incidence of 1 per 1,000 translates to 17,000 new cases per year. The societal impact of such an incidence of neuralgic amyotrophy would be quite significant. In the Netherlands, with a workforce estimated at 7.1 million, lost labor productivity related to this condition could cause the potential loss of USD 40,000,000 per year. This would assume a loss of USD 100 per day (240 days per year) per each affected employee, with approximately 25% of affected employess being unable to return to work. [2,6].

Our findings suggest that neither sex nor onset age are helpful in distinguishing neuralgic amyotrophy patients from patients with other disorders of the neck, shoulder and arms. However, when patients present with a typical history of acute severe pain, it probably is helpful to assess them for weakness, sensory symptoms, pain localization at onset, and when present the specific type of antecedent event. Points that help making the diagnosis are summed up in Table 4. Contrary to earlier assumptions, a limited passive range of glenohumeral motion is also often present as a complication in patients with definite neuralgic amyotrophy. This most likely reflects the strong association between scapulothoracic dyskinesia and the risk of glenohumeral complications [4].

**Table 4:** Points that help making the correct diagnosis of neuralgic amyotrophy.

*Consider*:
• Any patient with acute onset of very severe (NRS ≥ 7/10), analgesic resistent shoulder and/or upper arm pain
• Pain often worse at night and also severe when arm is at rest
• Multifocal peripheral nervous system symptoms and signs that can be bilateral but asymmetric
*Test*:
• Inspect and palpate shoulder with upper body and arms bared for scapular asymmetry and muscle atrophy
• Look for scapular dyskinesia from dorsal viewpoint with one slow shoulder abduction—anteflexion and vice versa movement (Some video examples of this type of movement can be seen following the links on this Radboudumc website page: https://www.radboudumc.nl/Zorg/Ziektebeelden/Pages/neuralgischeamyotrofie.aspx)
• Test and compare bilateral strength of serratus anterior, shoulder exorotation, long thumb and index finger flexors and forearm pronation: any weakness found in a combination of these is suspect for NA and rare in other disorders with similar presentations

The strong points of our study are that we prospectively counted all new episodes of neck, shoulder and arm complaints in a primary care setting using a mandatory coding system for each visit. From these cases the general practitioners were able to identify the patients suspected of having neuralgic amyotrophy by a relatively low-cost and time efficient combination, of two one-hour teaching sessions followed by a simple diagnostic protocol to make the diagnosis. With these instructions the general practitioners identified three times more patients with suspected neuralgic amyotrophy than the number in whom the diagnosis was confirmed by an experienced neurologist, so we think the likelihood of missing further patients was low. All definite neuralgic amyotrophy patients fulfilled the available criteria for neuralgic amyotrophy and only patients with the typical classic phenotype of (at least) periscapular weakness and a pain score of 7 or higher were included, leaving as little room for diagnostic uncertainty as possible.

Limitations of our study are that all practitioners involved were aware of the study goal, which could have led to an ascertainment bias. However, of the 34 patients referred for confirmation, the diagnosis neuralgic amyotrophy was rejected 12 times and any case with uncertainty or insufficient information was excluded from the definite count. We also did not systematically refer every patient with suspected neuralgic amyotrophy for neurological examination or ancillary investigations. However, neuralgic amyotrophy is primarily a clinical diagnosis made by adhering to clinical criteria [18] and there is no other gold-standard test to confirm or disprove it. Ancillary investigations primarily help to confirm or exclude the (co-) existence of other disorders. They can be (false-)negative in quite a number of neuralgic amyotrophy patients (nerve conduction studies) or show abnormalities such as cervical degenerative pathology (cervical spine MRI) that do not explain the clinical phenotype [2,21].

To conclude, the incidence of neuralgic amyotrophy appears to be much higher than previously thought. We suggest that raising awareness of the disorder in the primary care setting, combined with a short educational program for general practitioners on how to diagnose, and, when suspected, refer to a neurologist for confirmation, can help to quickly and accurately identify these patients. This will pave the way for acute phase immunomodulating therapy trials and hopefully the early prevention of complications [4,22] that now affect the majority of neuralgic amyotrophy patients on a long term basis.

## Supporting Information

S1 Case Descriptions Clinical detail overview of the patients who fulfilled the criteria for definite neuralgic amyotrophy. (PDF)

S1 Datafile Anonymized primary study data file in SPSS (IBM SPSS Statistics v20) format. (SAV)
