# Supplementary material for: Incidence of Neuralgic Amyotrophy (Parsonage Turner Syndrome) in a Primary Care Setting - A Prospective Cohort Study
Source: PLoS One. 2015 May 27;10(5):e0128361. doi: 10.1371/journal.pone.0128361 (PMC4445915; doi:10.1371/journal.pone.0128361)
Supplement: S1 Case Descriptions — (PDF) [file pone.0128361.s001.pdf]

Appendix. Case descriptions of definite neuralgic amyotrophy patients.

Case #060
Male, 55 years. Acute severe pain left shoulder (NRS 9) with weakness of left hand and paresthesia in digits I-III after 10 days. Little pain improvement with an NSAID, tramadol or long-acting opioid. No corticosteroid treatment. Arm feels heavy when lifting. Some improvement after 3 months but exertional myalgia of the left shoulder. Despite regular physiotherapy this was still present on follow up after 1 year. Examination showed left scapular winging on arm elevation, weakness of serratus anterior MRC grade 3, finger extensors, squeezing and thumb abduction MRC grade 4. No sensory disturbances, normal tendon reflexes.

Case #077
Female, 36 years. Acute severe pain (NRS 9) left shoulder with weakness shoulder abduction, shoulder exorotation and forearm pronation on the left after 2 weeks. No pain improvement with tramadol, slight improvement with long-acting opioid. No corticosteroid treatment. In month previous some mild interscapular pains, no specific antecedent event. Negative family history. On follow up a year later some strength improvement but the arm was still painful with use. Physiotherapy had had no effect. Examination showed left scapular dyskinesia with serratus anterior weakness (MRC grade 4) and overcompensating, painful trapezius and levator scapulae. Left shoulder exorotation MRC grade 4, latissimus dorsi MRC grade 4. No sensory disturbance, normal tendon reflexes.

Case #079
Male, 47 years. Acute severe pain left shoulder (NRS 9-10) lasting for weeks. After a few days weakness left arm and pain, weakness and sensory disturbances in left ventral upper leg. Treated with oral prednisolone 60 mg for 7 days, without clear effect. Tramadol 100 mg for 10 days gave some pain relief. No antecedent event, no family history. On follow up after 18 months still left shoulder pain NRS 4-5, with bilateral scapulothoracic dyskinesia and passive ROM restriction of the left glenohumeral joint. Atrophy of left supraspinatus and dorsal interosseus muscles. Weakness left serratus anterior, shoulder exorotation, shoulder abduction, triceps, finger extension and finger spreading MRC grade 4. Hypesthesia ventral left upper leg and decreased knee jerk on left.

Case #095
Male, 40 years. Some weeks after common cold and days after strenuous exercise development of sub acute severe (NRS 7) left shoulder pain. No analgesic or corticosteroid treatment. On follow up 2 months later a winged scapula and infraspinatus atrophy were seen, with weakness of left serratus anterior, shoulder exorotation, supraspinatus, biceps, triceps, forearm pronation and long finger and thumb flexors (MRC grade 4), paresthesia in median nerve area, diminished left triceps tendon reflex. Physiotherapy was started, with an

unclear effect. On final follow up after 18 months no more pain and 85% functional recovery, with remaining scapular dyskinesia and slight residual weakness of the infraspinatus and long finger flexors.

Case #104

Female, 30 years. Acute severe (NRS 10) right shoulder and arm pain 7-10 days postpartum. No analgesic or corticosteroid treatment. No passive ROM constraints but scapulothoracic dyskinesia and winged scapula, weakness right serratus anterior, shoulder exorotation, supraspinatus, deltoid, biceps brachii, triceps brachii, forearm pronation (MRC grade 4). No further treatment was initiated during the early phase. On follow up 11 months later no more pain and 90% functional recovery, but residual weakness and scapular dyskinesia still present on examination.

Case #135

Male, 62 years. Acute severe shoulder pain radiating in right arm, with improvement of pain over weeks. Afterwards exacerbations of pain (NRS 8) on movement. Temporary improvement on oral prednisone 60 mg for a week and tapering in the 2nd week, recurrent symptoms after weeks. No clear relief using diclofenac and pregabalin for 2 weeks. On follow up 3 months later still in pain, especially with arm abduction during work. Development of painful passive ROM right shoulder (frozen shoulder), scapulothoracic dyskinesia, weakness right serratus anterior and supraspinatus with supraspinatus atrophy. Regular physical therapy gave no improvement. Started on NA-specific rehabilitation program. On final follow up after a year no more pain except with severe overuse, regained 80% of previous functioning.

Case #156

Female, 41 years. After period of gradual increasing left arm pain acute increase to NRS 10. No effect of paracetamol. Oral prednisolone treatment 60 mg/day after several weeks with no clear effect. Scapular winging initially. No further treatment was initiated during the early phase. On follow up 1 year later a painful arc on the left, with left scapular dyskinesia and winging on shoulder abduction, hypesthesia in axillary nerve distribution.

Case #175

Male, 41 years. After 3 weeks of cervical pain acute increase with radiation to both shoulders and upper arm (NRS 8). Difficulty raising arms overhead and bilateral weakness of pinch grip. Oral long-acting opioid no clear effect on pain. High-dose oral prednisone 60 mg/day started by GP on day 2 with substantial pain resolution within 48 hours and minimal weakness improvement. Bilateral paresis of shoulder abduction MRC 2, elbow flexion and extension weakness more pronounced on right (MRC grade 4), right forearm pronation (MRC grade 4), forearm pronation on right (MRC grade 2), bilateral paralysis flexor indicis, superficial finger flexors MRC grade 2 on left, 0 on right. No sensory loss. Physiotherapy with

good effect, but on follow up 2 months later still scapular dyskinesia and substantial distal weakness with flexion contractures of the 1st metacarpophalangeal joints.

Case #206
Male, 33 years. Days after vaccination severe left shoulder pain (NRS 8) radiating to the back and chest. Diclofenac treatment no effect. Pain improved over weeks, but recurred weeks after. No further treatment was initiated during the early phase. On follow up 1 year later mild to moderate secondary pain of the left shoulder. Examination showed slight left scapulothoracic dyskinesia with scapular winging on anteflexion, MRC grade 4 weakness of left serratus anterior, infraspinatus, forearm pronation, common finger extensors and intrinsic hand muscles. Diminished biceps tendon reflex on the left.

Case #300
Male, 31 years. Acute severe pain upper arms 3 days after onset of high fever and proven Parvovirus B19 infection. Weakness of both hands and forearms with bilateral asymmetric scapular winging and atrophy of forearm muscles. Initial treatment with paracetamol, NSAID and tramadol no effect; slight pain improvement on long-acting opioid. Was started on oral prednisone after a week, with partial resolution of pain, but recurrence after tapering the steroids. Positive family history for NA (affected father and brother). Physiotherapy had a moderate effect, but on follow up 2 months later signs of overuse and myalgia of the affected muscles, with weakness of serratus anterior and pronator quadratus bilaterally MRC grade 2, long thumb flexors (left MRC grade 2, right grade 4) and bilateral wrist extensors. Hypesthesia in area lateral antebrachial cutaneous nerve.

Case #307
Female, 48 years. Acute pain right upper arm, in 3 days maximum pain (NRS 9-10). High-dose oral prednisone 60 mg/day for 1 week started on day 2, with full pain resolution in 48 hours but stiff awkward sensation in right arm remaining. No analgesics used. No antecedent event, negative family history. Started on regular physiotherapy but got worse. On follow up 3 months later still continuous right arm pain NRS 3-5, could not sleep on affected side. On examination right scapular dyskinesia with elevated and protracted shoulder, trapezius overuse and limited active range of motion with maximum 80 degrees abduction. Shoulder exorotation, forearm pronation, finger flexor and finger extensor weakness MRC grade 4 on right. Hypesthesia in axillary and lateral antebrachial cutaneous nerve distribution. Normal tendon reflexes.

Case #339
Male, 69 years. Acute very severe pain (NRS 10) left shoulder 5 days after uncomplicated left glenohumeral surgery, followed by mild scapular winging and deltoid atrophy. No effect of paracetamol, NSAID, tramadol, long-acting opioids or amitriptyline. No corticosteroid treatment. Weakness of left serratus anterior with scapulothoracic dyskinesia, MRC grade 4

weakness of subscapularis, triceps brachii, pronator quadratus, long thumb flexor and deep finger flexors of digits II-III. Atrophy of left infraspinatus and deltoid. Diminished left biceps and brachioradial tendon reflex. Regular physical therapy had no effect. Slow improvement over months with overall fatigue and decreased endurance with exertional myalgia of affected arm. On follow up also signs of left rotator cuff impingement.

Case #342
Female, 41 years. Mild neck pain for some weeks, acute severe right shoulder pain (NRS 8) at 4 AM and weakness within days. No effect of paracetamol, NSAID or long-acting opioid. Improvement on prednisolone 60 mg for a week with tapering in the 2nd week. No antecedent event. No passive ROM constraints, minimally disturbed scapulothoracic rhythm, subtle scapular winging. No physical therapy started. On follow up 1 year later still weakness of right serratus anterior, triceps brachii, long thumb flexor and and deep finger flexors of digits II-III MRC grade 4. Hypesthesia right digits I and II and diminished triceps tendon reflex.

Case #411
Male, 44 years. Acute pain (NRS 9-10) right neck, shoulder and arm at 3 AM. Weakness of shoulder and hand muscles bilaterally with winging scapula within days. No autonomic symptoms. Initial pain duration 6 weeks, with no clear improvement on oral prednisolone 60 mg/day for 10 days and only slight pain reduction with combination of NSAID and oral long-acting opioid. Paresis slowly improving over the course of a year with subsiding pain. Physiotherapy had no effect. On follow up after 18 months still moderately severe pain of the left shoulder, works 28 out of his previous 40 hours a week. No passive ROM constraints but scapulothoracic dyskinesia on the right with shoulder winging on abduction, weakness serratus anterior right > left, infraspinatus bilaterally, left pronator teres, both pronator quadratus and long finger flexors, hand intrinsics and left finger extensors, all MRC grade 4.
